# 3-Methyl­benzene-1,2-diamine

**DOI:** 10.1107/S1600536812046120

**Published:** 2012-11-14

**Authors:** Xiao-Li Yang, Zhi-Qiang Feng, Ling-Yun Hao

**Affiliations:** a School of Material Engineering, Jinling Institute of Technology, Nanjing 211169, People’s Republic of China

## Abstract

The title compound, C_7_H_10_N_2_, was synthesized from 2-methyl-6-nitro­aniline by a reduction reaction. In the crystal, molecules are linked *via* N—H⋯N hydrogen bonds, forming two-dimensional networks lying parallel to (100). These networks are stabilized by C—H⋯π and N—H⋯π inter­actions.

## Related literature

The title compound is an important organic synthesis inter­mediate. For background to its applications, see: Wen *et al.* (2009 ▶). For the synthetic procedure, see: Li *et al.* (2011 ▶). For bond-length data, see: Allen *et al.* (1987 ▶).
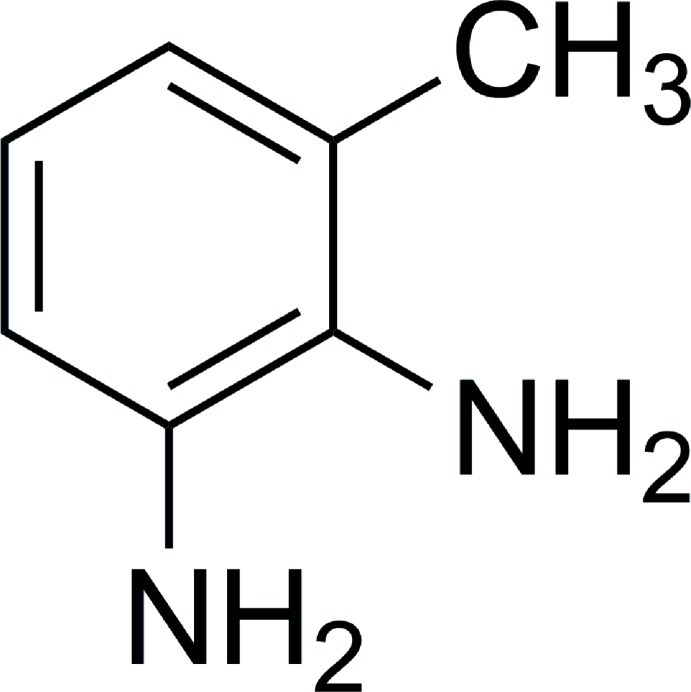


## Experimental

### 

#### Crystal data

C_7_H_10_N_2_
*M* *_r_* = 122.17
Monoclinic, 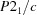

*a* = 11.836 (2) Å
*b* = 7.7160 (15) Å
*c* = 7.7430 (15) Å
β = 90.72 (3)°
*V* = 707.1 (2) Å^3^
*Z* = 4
Mo *K*α radiation
μ = 0.07 mm^−1^
*T* = 291 K
0.3 × 0.2 × 0.1 mm

#### Data collection

Enraf–Nonius CAD-4 diffractometer
Absorption correction: ψ scan (North *et al.*, 1968 ▶) *T*_min_ = 0.979, *T*_max_ = 0.993
2693 measured reflections
1300 independent reflections
962 reflections with *I* > 2σ(*I*)
*R*_int_ = 0.060
3 standard reflections every 200 reflections intensity decay: 1%

#### Refinement

*R*[*F* ^2^ > 2σ(*F* ^2^)] = 0.045
*wR*(*F* ^2^) = 0.124
*S* = 1.00
1300 reflections
110 parameters
H atoms treated by a mixture of independent and constrained refinement
Δρ_max_ = 0.13 e Å^−3^
Δρ_min_ = −0.12 e Å^−3^

### 

Data collection: *CAD-4 Software* (Enraf–Nonius, 1985 ▶); cell refinement: *CAD-4 Software*; data reduction: *XCAD4* (Harms & Wocadlo,1995 ▶); program(s) used to solve structure: *SHELXS97* (Sheldrick, 2008 ▶); program(s) used to refine structure: *SHELXL97* (Sheldrick, 2008 ▶); molecular graphics: *SHELXTL* (Sheldrick, 2008 ▶); software used to prepare material for publication: *SHELXTL*.

## Supplementary Material

Crystal structure: contains datablock(s) I, global. DOI: 10.1107/S1600536812046120/bq2378sup1.cif

Structure factors: contains datablock(s) I. DOI: 10.1107/S1600536812046120/bq2378Isup2.hkl

Supplementary material file. DOI: 10.1107/S1600536812046120/bq2378Isup3.cml

Additional supplementary materials:  crystallographic information; 3D view; checkCIF report

## Figures and Tables

**Table 1:** Hydrogen-bond geometry (Å, °) *Cg*1 is the centroid of C1–C6 ring.

*D*—H⋯*A*	*D*—H	H⋯*A*	*D*⋯*A*	*D*—H⋯*A*
N1—H1*A*⋯N2^i^	0.91 (2)	2.36 (2)	3.237 (2)	163.5 (16)
N2—H2*A*⋯N1^ii^	0.84 (2)	2.48 (2)	3.248 (2)	152.2 (19)
C2—H2⋯*Cg*1^iii^	0.93	2.85	3.6713 (18)	148
N2—H2*B*⋯*Cg*1^iv^	0.90 (2)	2.776 (18)	3.5192 (17)	141.2 (16)

Symmetry codes: (i) 
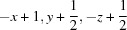
; (ii) 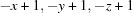; (iii) 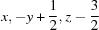; (iv) 
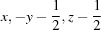
.

**Fig. 1.**
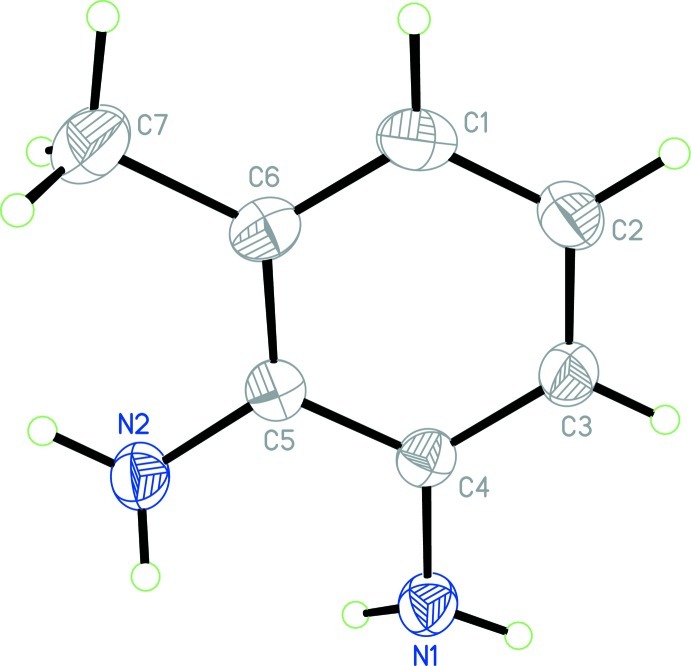
The molecular structure of (I), with the atom-numbering scheme. Displacement ellipsoids are drawn at the 50% probability level.

**Fig. 2.**
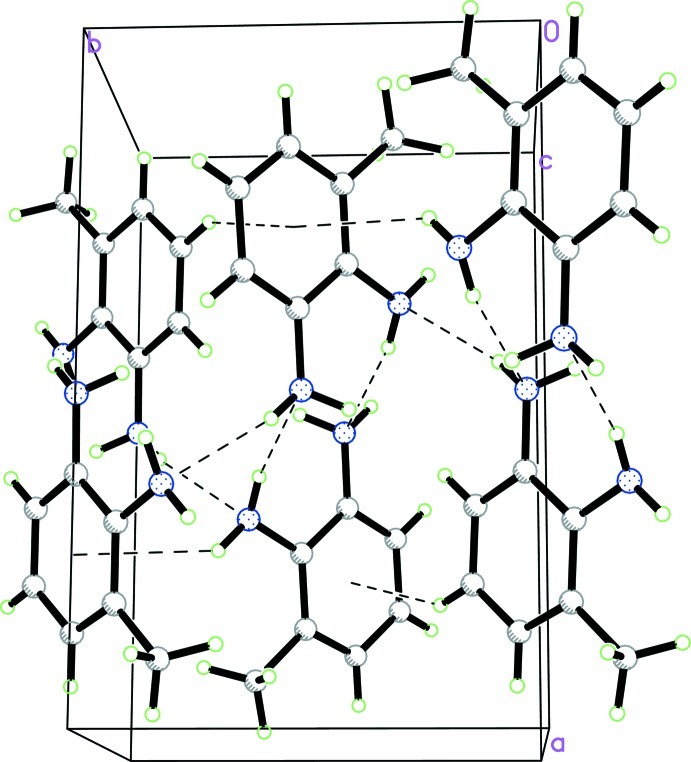
A packing diagram of (I). Hydrogen bonds are shown by dashed lines.

## supplementary crystallographic information

### Comment

Aromatic amines are important organic intermediates and widely used in chemical, pharmaceutical, agricultural and photographic chemicals. Aromatic amines are mainly synthesized by the reduction of aromatic nitro compounds. Compared with other reduction methods, catalytic transfer hydrogenation (CTH) is environmentally friendly, high yield and good selectivity (Wen *et al.*, 2009), in which hydrogen gas is replaced by a hydrogen donor such as hydrazine hydrate. Here, the title compound, (I), was synthesized by the CTH of 2-methyl-6-nitroaniline, and we report the crystal structure of (I).

In the molecule of (I), (Fig.1), the bond lengths (Allen *et al.*, 1987) and angles are within normal ranges. In the crystal, there are two C—H···*Cg*1 and N—H···*Cg*1 interactions (Cg1 is the centroid of C1-C6 ring). The molecules are linked each other by the two intermolecular N—H···N hydrogen bonds to form a three-dimensional network, which seem to be very effective in the stabilization of the crystal structure (Fig. 2.).

### Experimental

The title compound, (I) was prepared by the literature method (Li *et al.*, 2011). Crystals suitable for X-ray analysis were obtained by dissolving (I) (0.5 g) in ethyl acetate (20 ml) and evaporating the solvent slowly at room temperature for about 7 d.

### Refinement

H atoms were positioned geometrically, with C—H = 0.93 Å for aromatic H, and constrained to ride on their parent atoms, with *U*_iso_(H) = *xU*_eq_(C/N), where *x* = 1.2 for aromatic H.

### Crystal data

**Table:**

C_7_H_10_N_2_	*F*(000) = 264
*M**_r_* = 122.17	*D*_x_ = 1.148 Mg m^−^^3^
Monoclinic, *P*2_1_/*c*	Mo *K*α radiation, λ = 0.71073 Å
Hall symbol: -P 2ybc	Cell parameters from 25 reflections
*a* = 11.836 (2) Å	θ = 9–13°
*b* = 7.7160 (15) Å	µ = 0.07 mm^−^^1^
*c* = 7.7430 (15) Å	*T* = 291 K
β = 90.72 (3)°	Needle, colourless
*V* = 707.1 (2) Å^3^	0.3 × 0.2 × 0.1 mm
*Z* = 4	

### Data collection

**Table:**

Enraf–Nonius CAD-4 diffractometer	962 reflections with *I* > 2σ(*I*)
Radiation source: fine-focus sealed tube	*R*_int_ = 0.060
Graphite monochromator	θ_max_ = 25.4°, θ_min_ = 1.7°
ω/2θ scans	*h* = −14→14
Absorption correction: ψ scan (North *et al.*, 1968)	*k* = −9→9
*T*_min_ = 0.979, *T*_max_ = 0.993	*l* = 0→9
2693 measured reflections	3 standard reflections every 200 reflections
1300 independent reflections	intensity decay: 1%

### Refinement

**Table:**

Refinement on *F*^2^	Primary atom site location: structure-invariant direct methods
Least-squares matrix: full	Secondary atom site location: difference Fourier map
*R*[*F*^2^ > 2σ(*F*^2^)] = 0.045	Hydrogen site location: inferred from neighbouring sites
*wR*(*F*^2^) = 0.124	H atoms treated by a mixture of independent and constrained refinement
*S* = 1.00	*w* = 1/[σ^2^(*F*_o_^2^) + (0.073*P*)^2^] where *P* = (*F*_o_^2^ + 2*F*_c_^2^)/3
1300 reflections	(Δ/σ)_max_ < 0.001
110 parameters	Δρ_max_ = 0.13 e Å^−^^3^
0 restraints	Δρ_min_ = −0.12 e Å^−^^3^

### Special details

**Table:**

Geometry. All e.s.d.'s (except the e.s.d. in the dihedral angle between two l.s. planes) are estimated using the full covariance matrix. The cell e.s.d.'s are taken into account individually in the estimation of e.s.d.'s in distances, angles and torsion angles; correlations between e.s.d.'s in cell parameters are only used when they are defined by crystal symmetry. An approximate (isotropic) treatment of cell e.s.d.'s is used for estimating e.s.d.'s involving l.s. planes.
Refinement. Refinement of *F*^2^ against ALL reflections. The weighted *R*-factor *wR* and goodness of fit *S* are based on *F*^2^, conventional *R*-factors *R* are based on *F*, with *F* set to zero for negative *F*^2^. The threshold expression of *F*^2^ > σ(*F*^2^) is used only for calculating *R*-factors(gt) *etc*. and is not relevant to the choice of reflections for refinement. *R*-factors based on *F*^2^ are statistically about twice as large as those based on *F*, and *R*- factors based on ALL data will be even larger.

### Fractional atomic coordinates and isotropic or equivalent isotropic displacement parameters (Å^2^)

**Table:**

	*x*	*y*	*z*	*U*_iso_*/*U*_eq_	
C1	0.15863 (14)	0.55588 (19)	0.1128 (2)	0.0551 (5)	
H1	0.0829	0.5612	0.0799	0.066*	
C2	0.23534 (15)	0.6592 (2)	0.0300 (2)	0.0578 (5)	
H2	0.2116	0.7340	−0.0573	0.069*	
C3	0.34751 (13)	0.65096 (18)	0.07727 (19)	0.0493 (4)	
H3	0.3996	0.7206	0.0211	0.059*	
C4	0.38402 (12)	0.54018 (16)	0.20768 (17)	0.0408 (4)	
C5	0.30472 (12)	0.43631 (17)	0.29360 (17)	0.0399 (4)	
C6	0.19124 (12)	0.44367 (18)	0.2446 (2)	0.0478 (4)	
C7	0.10562 (18)	0.3339 (3)	0.3346 (3)	0.0760 (6)	
N1	0.49730 (11)	0.5349 (2)	0.26271 (18)	0.0508 (4)	
N2	0.34528 (14)	0.32174 (18)	0.42184 (19)	0.0525 (4)	
H1A	0.5472 (17)	0.596 (3)	0.199 (3)	0.072 (6)*	
H1B	0.5231 (14)	0.430 (3)	0.290 (2)	0.066 (6)*	
H2A	0.4043 (19)	0.350 (3)	0.478 (3)	0.086 (7)*	
H2B	0.2929 (18)	0.257 (2)	0.474 (3)	0.074 (6)*	
H7A	0.031 (2)	0.350 (3)	0.279 (3)	0.097 (7)*	
H7B	0.127 (2)	0.211 (3)	0.323 (3)	0.111 (8)*	
H7C	0.1046 (18)	0.375 (3)	0.458 (3)	0.091 (7)*	

### Atomic displacement parameters (Å^2^)

**Table:**

	*U*^11^	*U*^22^	*U*^33^	*U*^12^	*U*^13^	*U*^23^
C1	0.0485 (9)	0.0534 (9)	0.0630 (10)	0.0031 (7)	−0.0144 (8)	−0.0054 (8)
C2	0.0675 (11)	0.0475 (9)	0.0578 (10)	−0.0018 (8)	−0.0169 (8)	0.0077 (8)
C3	0.0583 (10)	0.0435 (8)	0.0461 (9)	−0.0086 (7)	−0.0023 (7)	0.0014 (7)
C4	0.0458 (8)	0.0359 (7)	0.0408 (8)	−0.0009 (6)	0.0020 (6)	−0.0065 (6)
C5	0.0445 (8)	0.0354 (7)	0.0398 (7)	0.0032 (6)	0.0025 (6)	−0.0045 (6)
C6	0.0424 (8)	0.0444 (8)	0.0566 (9)	−0.0021 (6)	0.0008 (7)	−0.0062 (7)
C7	0.0493 (11)	0.0862 (16)	0.0926 (17)	−0.0115 (10)	0.0066 (11)	0.0150 (13)
N1	0.0437 (8)	0.0531 (9)	0.0558 (8)	−0.0016 (6)	0.0033 (6)	0.0063 (7)
N2	0.0482 (8)	0.0534 (8)	0.0557 (8)	0.0009 (7)	0.0021 (7)	0.0128 (7)

### Geometric parameters (Å, º)

**Table:**

C1—C2	1.373 (2)	C5—N2	1.4092 (19)
C1—C6	1.389 (2)	C6—C7	1.499 (2)
C1—H1	0.9300	C7—H7A	0.98 (2)
C2—C3	1.374 (2)	C7—H7B	0.99 (2)
C2—H2	0.9300	C7—H7C	1.01 (2)
C3—C4	1.388 (2)	N1—H1A	0.91 (2)
C3—H3	0.9300	N1—H1B	0.89 (2)
C4—N1	1.4023 (19)	N2—H2A	0.84 (2)
C4—C5	1.408 (2)	N2—H2B	0.90 (2)
C5—C6	1.392 (2)		
			
C2—C1—C6	121.70 (15)	C1—C6—C5	118.94 (14)
C2—C1—H1	119.2	C1—C6—C7	120.69 (16)
C6—C1—H1	119.2	C5—C6—C7	120.37 (15)
C1—C2—C3	119.44 (15)	C6—C7—H7A	109.4 (13)
C1—C2—H2	120.3	C6—C7—H7B	108.7 (16)
C3—C2—H2	120.3	H7A—C7—H7B	108.3 (19)
C2—C3—C4	120.86 (15)	C6—C7—H7C	106.2 (12)
C2—C3—H3	119.6	H7A—C7—H7C	110.6 (19)
C4—C3—H3	119.6	H7B—C7—H7C	113 (2)
C3—C4—N1	121.78 (14)	C4—N1—H1A	116.5 (12)
C3—C4—C5	119.42 (14)	C4—N1—H1B	115.0 (11)
N1—C4—C5	118.72 (13)	H1A—N1—H1B	112.1 (17)
C6—C5—C4	119.64 (13)	C5—N2—H2A	118.1 (14)
C6—C5—N2	122.45 (14)	C5—N2—H2B	115.9 (12)
C4—C5—N2	117.83 (14)	H2A—N2—H2B	118.8 (19)
			
C6—C1—C2—C3	0.5 (2)	N1—C4—C5—N2	−5.00 (19)
C1—C2—C3—C4	−0.2 (2)	C2—C1—C6—C5	0.1 (2)
C2—C3—C4—N1	−177.35 (13)	C2—C1—C6—C7	179.18 (17)
C2—C3—C4—C5	−0.6 (2)	C4—C5—C6—C1	−0.9 (2)
C3—C4—C5—C6	1.19 (19)	N2—C5—C6—C1	−177.77 (13)
N1—C4—C5—C6	178.00 (12)	C4—C5—C6—C7	179.99 (16)
C3—C4—C5—N2	178.19 (12)	N2—C5—C6—C7	3.1 (2)

### Hydrogen-bond geometry (Å, º)

Cg1 is the centroid of C1–C6 ring.

**Table:**

*D*—H···*A*	*D*—H	H···*A*	*D*···*A*	*D*—H···*A*
N1—H1*A*···N2^i^	0.91 (2)	2.36 (2)	3.237 (2)	163.5 (16)
N2—H2*A*···N1^ii^	0.84 (2)	2.48 (2)	3.248 (2)	152.2 (19)
C2—H2···*Cg*1^iii^	0.93	2.85	3.6713 (18)	148
N2—H2*B*···*Cg*1^iv^	0.90 (2)	2.776 (18)	3.5192 (17)	141.2 (16)

Symmetry codes: (i) −*x*+1, *y*+1/2, −*z*+1/2; (ii) −*x*+1, −*y*+1, −*z*+1; (iii) *x*, −*y*+1/2, *z*−3/2; (iv) *x*, −*y*−1/2, *z*−1/2.
